# Dosimetric analysis reveals rapid clearance and low absorbed dose of [¹⁷⁷Lu]Lu-PSMA-617 in non-prostate cancers with high PSMA expression

**DOI:** 10.1186/s13550-026-01394-z

**Published:** 2026-02-24

**Authors:** Fadi Khreish, Florian Rosar, Caroline Burgard, Sven Petto, Stephan Maus, Tobias Stemler, Mark Bartholomä, Amir Sabet, Andrea Schaefer-Schuler, Samer Ezziddin

**Affiliations:** 1 Dept. of Nuclear Medicine, Saarland University, Homburg, Germany; 2 Dept. of Nuclear Medicine, Klinikum Fulda, University of Marburg, Campus Fulda, Fulda, Germany; 3 Dept. of Nuclear Medicine, Frankfurt University, Frankfurt am Main, Germany; 4 Department of Nuclear Medicine, Marburg University, Campus Fulda Pacelliallee 4, D-36043 Fulda, Germany

**Keywords:** Dosimetry, [^177^Lu]Lu-PSMA-617, Radioligand therapy, RLT, Non-prostate malignancy

## Abstract

**Background:**

The aim of this study was to evaluate the dosimetry for [177Lu]Lu-PSMA-617 in advanced non-prostate cancer (non-PCa) patients with previous intense radiotracer uptake of the tumor lesions on PET/CT using [68Ga]Ga-PSMA-11.

**Results:**

Dosimetry data of 5 patients with non-prostate cancer (non-PCa group) were assessed and compared; Non-PCa tumors were breast cancer (BC), renal cell carcinoma (RCC), hepatocellular carcinoma (HCC) and anaplastic astrocytoma (AA). Five patients with metastatic castration-resistant prostate cancer (PCa group) were used as control-group. All patients were given [177Lu]Lu-PSMA-617 after proven sufficient PSMA uptake of tumor lesions by [68Ga]Ga-PSMA-11 PET/CT. Post-therapeutic dosimetry with serial whole-body scans (24, 48 and 72–120 h post-injection) included calculation of effective half-life and absorbed doses for tumor and non-tumor lesions and comparison between non-PCa and PCa patients. The mean effective half-life in tumor lesions was significantly shorter in non-PCa compared to PCa patients (27.1 ± 13.1 h vs. 74.9 ± 23.1 h, respectively, *p* < 0.001). Likewise, the mean absorbed dose per injected activity was significantly lower in tumor lesions of non-PCa compared to PCa (0.49 ± 0.40 Gy/GBq vs. 3.51 ± 2.20 Gy/GBq, respectively, *p* < 0.001). No significant differences for the source organ absorbed dose or effective half-life were observed between both groups.

**Conclusions:**

In non-prostate malignancy with impressive diagnostic PSMA-mediated tumor uptake, i.e. high tracer uptake at early time points in [68Ga]Ga-PSMA-11 PET/CT, [177Lu]Lu-PSMA-617 delivers a low tumor-absorbed dose due to a short effective half-life, therefore this therapy does not appear to be a potential antitumor option.

## Introduction

Prostate-specific membrane antigen (PSMA) is a type II membrane glycoprotein with an intracellular segment, a trans-membrane domain, and an extensive extracellular domain [1]. PSMA is overexpressed on prostate carcinoma cells [2]. The density of PSMA expression further increases dependent on the Gleason score, and castration-resistance, i.e. with increasing aggressiveness of prostate cancer [3]. The function of PSMA is currently unclear, but it is thought to be related to transmembrane transport mechanism. The property of PSMA makes it an ideal target for radionuclide imaging and therapy [4]. Radioligand therapy (RLT) targeting PSMA using PSMA radioligands labeled with the beta emitter Lutetium-177 (^177^Lu) has shown excellent results and has recently been FDA-approved as a treatment option for men with late-stage metastatic castration-resistant prostate cancer [5–7].

Despite its name, PSMA is not specific to the prostate cells but is also expressed in other normal tissues (e.g. in proximal tubules of the kidney, jejunal brush border of the small bowl, and salivary glands) and in several different malignant solid tumors like salivary gland carcinoma, lung cancer, breast cancer, hepatocellular carcinoma, renal cell carcinoma, thyroid carcinoma and glioblastoma [8–15]. PSMA is also highly expressed in the tumor-associated neovasculature of most solid malignancies [8, 16]. The promising results of [^177^Lu]Lu-PSMA-617 RLT in prostate cancer encouraged us to evaluate this treatment option in non-prostate cancer patients.

The aim of this study was to evaluate the radiation dosimetry of [^177^Lu]Lu-PSMA-617 in patients with non-prostate cancers who had a progressive disease after exhaustion of individual appropriate conventional therapies for each tumor entity and showed intense PSMA-positive tumor lesions on screening with [^68^Ga]Ga-PSMA-11 PET/CT.

## Methods

### Patient characteristics

In this retrospective study, dosimetry data obtained from *n* = 10 patients treated with [^177^Lu]Lu-PSMA-617 RLT were analyzed. The patient cohort consists of five non-prostate cancer patients (non-PCa group), of which two had breast cancer (BC), one had hepatocellular carcinoma (HCC), one had renal cell carcinoma (RCC) and one had anaplastic astrocytoma (AA). Additionally, five patients with metastatic castration-resistant prostate cancer (PCa) were included in this study as a control group (PCa group). The details of the patient cohort are given in Table 1. All patients showed disease progression after exhaustion of conventional therapy options of each tumor entity. Furthermore, all patients showed intense PSMA expressing metastases or recurrence disease on screening by [^68^Ga]Ga-PSMA-11 PET/CT. Intense PSMA expression, which was required for decision-making, was defined as tracer uptake that was higher than physiologic uptake of the healthy liver tissue. To exclude patients with PSMA-negative tumor lesions, ^18^F-fluorodeoxyglucose (FDG)-PET/CT was performed in all patients except the patient with anaplastic astrocytoma, who underwent ^18^F-fluoroethyl-L-tyrosine (FET)-PET/CT imaging. The decision to offer [^177^Lu]Lu-PSMA-617 RLT was made by the local interdisciplinary institutional tumor board. Patients were informed in detail about the highly experimental character of this therapy and gave written informed consent. The study was performed in accordance with the Declaration of Helsinki and in particular with regard to the German Pharmaceutical Act § 13 (2b).

**Table 1:** Baseline characteristics of the all patients

Patient	Sex	Age	Tumor-Entity	[^177^Lu]Lu-PSMA-617 activity (GBq)	Prior anti-tumor therapies
1.	F	54	BC	2.7	Resection; Docetaxel; Tamoxifen; Letrozol; Paclitaxel/Bevacizumab; Eribulin; ^153^Sm therapy; Doxorubicin; Carboplatin/Gemcitabine
2.	F	78	BC	5.8	Adjuvant external radiation; Aromasin; Tamoxifen; ^153^Sm therapy; Fulvestrant
3.	M	45	HCC	4.4	Sorafinib; 2x Radioembolization (SIRT); external radiation (bone metastases)
4.	F	83	RCC	4.0	Tumor-nephrectomy; Stabilization and external radiation of bone metastases
5.	M	73	AA	6.4	Resection; external radiation + Temodal; PCV-(Procarbazin, CCNU, Vincristin) Schema; proton-radiation therapy; Bevacizumab + Irinotecan
6.	M	91	PCa	7.3	Orchiectomy; external Radiation; ADT; Abiraterone; Enzalutamide; Docetaxel
7.	M	66	PCa	6.1	ADT; Abiraterone; ^223^Ra therapy; Docetaxel; Enzalutamide
8.	M	68	PCa	8.9	ADT; Docetaxel; Abiraterone; ^223^Ra therapy
9.	M	81	PCa	8.9	External radiation; ADT; Abiraterone; Enzalutamide
10.	M	68	PCa	9.7	Docetaxel; ADT; Abiraterone; Enzalutamide; Cabazitaxel

BC, breast cancer; HCC, hepatocellular carcinoma; SIRT, selective internal radiation therapy; RCC, renal cell carcinoma; PCa, prostate cancer; ADT, androgen-deprivation therapy

### Preparation of [^177^Lu]Lu-PSMA-617

PSMA-617 precursor was obtained from ABX advanced biochemical compounds GmbH, Radeberg (Germany) and ^177^LuCl_3_ from IDB Holland BV, Baarle-Nassau (The Netherlands). The production and quality control of [^177^Lu]Lu-PSMA-617 was accomplished in analogy to the previously published methodology in literature [17]. For a typical labelling 150 µg (143 nmol) PSMA-617 were used for 6 GBq of ^177^Lu. The radiochemical yield and purity were > 99%.

### [^68^Ga]Ga-PSMA-11 PET/CT

All PET/CT studies were performed on a Biograph mCT 40 scanner (Siemens Medical Solutions, Knoxville, TN, USA) comprising whole-body PET/CT scans extending from vertex to mid-femur in 3D mode. [^68^Ga]Ga-PSMA-11 was intravenously injected with a mean injected activity of 107 ± 26 MBq (range: 75–136 MBq) followed by a 500 ml infusion of 0.9% NaCl. The image acquisition started about 60 min after injection of the tracer, in accordance with standard procedures for prostate cancer imaging [18]. PET acquisition-time was 3 min per bed position, image matrix size 200 × 200. CT acquisition was performed in low-dose technique using an X-ray tube voltage of 120 keV and a modulation of the tube current (CARE Dose4D, Siemens Erlangen; maximal tube current: 30 mA) followed by reconstruction with a soft tissue reconstruction kernel (B31f) to a slice thickness of 5 mm (increment: 2–4 mm). PET emission datasets were corrected for decay, randoms and scatter and reconstructed using an iterative 3-dimensional ordered subset expectation maximization algorithm (3 iterations; 24 subsets) with gaussian filtering to a transaxial resolution of 5 mm. Attenuation correction was performed using the non-enhanced low-dose CT-data.

### [^177^Lu]Lu-PSMA-617 RLT

The mean administered activity of [^177^Lu]Lu-PSMA-617 was 4.6 ± 1.5 GBq (range: 2.7–6.4 GBq) in non-PCa patients and 8.2 ± 1.5 GBq (range: 6.1–9.7 GBq) in PCa patients. Each patient of the non-PCa group was treated with one cycle of [^177^Lu]Lu-PSMA-617. The patients of PCa group were treated with several cycles of [^177^Lu]Lu-PSMA-617. However, only the results of the first cycle were included in this analysis. The treatments were performed during in-patient stay according to national radiation protection regulations. Each patient received intravenous hydration (1 l of 0.9% NaCl) starting 30 min before therapy. The radiopharmaceutical was administered intravenously over 15–20 min. Prior and concomitant to the radiopharmaceutical administration the salivary glands were cooled with ice packs starting 30 min before radiopharmaceutical infusion, lasting until 2 h post-infusion. All patients were clinically monitored during the entire hospitalization for possible side effects including vital signs.

### Dosimetry with planar whole-body imaging

All post-therapeutic imaging was performed on a dual headed Bright View SPECT/CT gamma camera system (Philips Medical Systems, Hamburg, Germany) equipped with medium-energy parallel-hole collimators and opposing (180° mode) detector heads. For the purpose of this dosimetry study, patients underwent posttreatment serial planar whole body (WB) imaging. WB scans were acquired 1, 2 and 3–5 d (approx. 24 h, 48 h, and ≥ 72 h p.i.) post-injection. A ^177^Lu-reference vial was measured simultaneously for calibration purposes. The energy window was set to 208 keV with a width of 20% as proposed in previous studies [19–21]. A respective low scatter window (187 keV, 15%) was defined in order to perform scatter correction on the WB images according to the dual-energy window technique proposed by MIRD (MIRD Pamphlet No. 16 [22]). For WB acquisition, the scanning speed was 15 cm/min on day 1 and day 2 and 12 cm/min on day ≥ 3. The matrix size was 256 × 1024, the pixel size 2.33 × 2.33 mm^2^. WB transmission and blank scans were acquired 1 d before therapy by use of a flat phantom filled with aqueous solution of ^177^Lu. The corresponding images were used to correct the planar whole-body data for attenuation.

### ***Radiatoni*** dosimetry Estimation

For radiation dosimetry estimation, the conjugate-view method [22] was used to calculate the source organ activities from the anterior-posterior WB images. In a first step, a scatter estimate image was derived from the adjacent scatter energy window with the dual-energy window method and subtracted from the respective emission image [22]. These scatter-corrected images were used for the dosimetry analysis which was performed by application of the QDOSE dosimetry software suite (ABX-CRO, Dresden, Germany). The target tumor lesions were selected based on SUV on PSMA-PET/CT and their location. We identified the most intense (highest SUV) lesions on PSMA-PET/CT and lesions that were as far as possible in the periphery to avoid overlap with physiological PSMA uptake in normal tissue. In QDOSE, all WB scintigrams including transmission images were manually co-registered. Boundary regions-of-interest (ROI) were manually drawn solely enclosing selected tumor lesions without overlap with other tissues of tracer accumulation. The kidneys, liver, salivary glands (both left and right parotid and submandibular glands) and total body were included as source organs if non-superimposed by tumor accumulation. For the RCC patient who had a nephrectomy, the values refer only to the remaining kidney. Boundary ROI of all segmented source organs were manually drawn in the image that allowed the best organ delineation and then were copied onto all other time points. Individual background ROIs were placed as close as possible to the respective target without superimposition by other activity sources. A threshold-based segmentation was applied in order to delineate the organ or tumor within the manually drawn boundary ROI taking into account the anterior as well as the posterior image. Calibration of the background-corrected ROI counts in relation to activity concentration was performed by use of the reference vial in combination with a calibration factor derived previously by phantom measurements [23]. The estimated activities were used to determine time–activity curves for each source organ, lesion and the total body by mono-exponential regression of the serial measurements using weighted least squares method and to estimate the respective time-integrated activities (TIA) and time-integrated activity coefficients (TIAC) by numerical integration. For the first-time interval, constant activity was assumed, and the integration method was based on the trapezoidal method. Between the first and the last time point, a trapezoidal integration was also used, whereas after the last measured time-point to infinity, analytical integration was performed considering the mono-exponential fitting curve.

Absorbed and effective dose calculations were performed using the International Commission on Radiological Protection (ICRP)–endorsed IDAC-Dose 2.1 [24], which is implemented in QDOSE. IDAC-Dose 2.1 is based on the ICRP adult reference computational phantoms [25] and the ICRP-specific absorbed fractions [26]. Organ masses were adapted to individual subject organ masses as extracted from the diagnostic CT scan acquired prior to therapy. To approximate the absorbed dose to the salivary glands and the lesions, the sphere S values of OLINDA were used which are also integrated in QDOSE. Organ masses for the salivary glands were taken from ICRP publication 89 with 25 g estimated weight for the parotid and 12.5 g for the submandibular gland [27].

## Statistics analysis

All continuous data reported are expressed as mean, SD, and range. For statistical analysis, Mann-Whitney U test was applied using Prism 8 (GraphPad Software, San Diego, USA) to determine significant differences between the groups. The significance level was *p* < 0.05.

## Results

### Pre- and post-therapeutic imaging

**Fig. 1:**
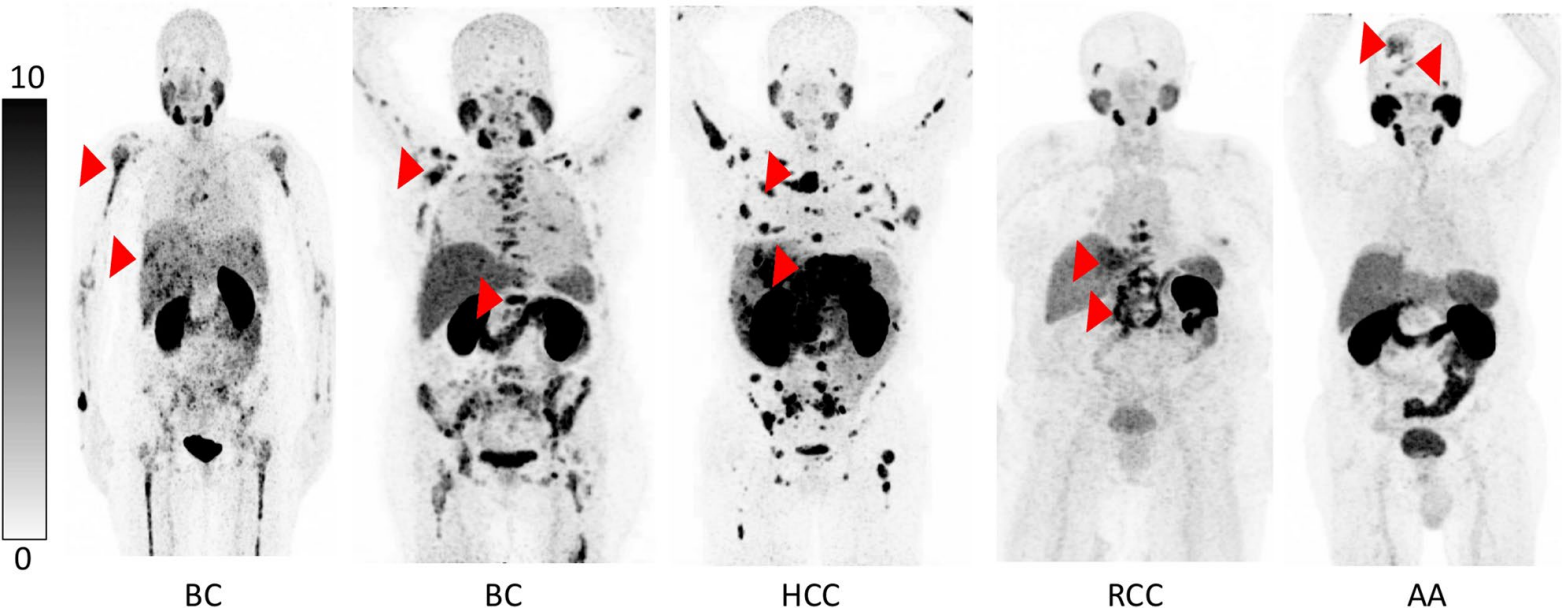
Maximum intensity projections (MIP) of screening [^68^Ga]Ga-PSMA-11 PET/CT in non-PCa patients. Red arrows point to exemplary tumor lesions. BC, breast cancer; HCC, hepatocellular carcinoma; RCC, renal cell carcinoma; AA, anaplastic astrocytoma

Data from different PSMA-positive non-prostate cancers were analyzed, including breast cancer (BC), hepatocellular carcinoma (HCC), renal cell carcinoma (RCC), and anaplastic astrocytoma (AA). All non-PCa patients included in this study showed an intense PSMA expression of tumor lesions on screening [^68^Ga]Ga-PSMA-11 PET/CT and were therefore treated with one cycle of [^177^Lu]Lu-PSMA-617 RLT. Figure 1 shows PET/CT images of all five non-PCa patients demonstrating the high PSMA expression of the tumor lesions. Mean SUVmax of target lesions in these patients was 15.8 ± 6.6 (range: 9.8–31.2). Comparing to the PCa group, mean SUVmax of target lesions in PCa patients was 34.9 ± 21.8 (range: 10.6–88.6).

**Fig. 2:**
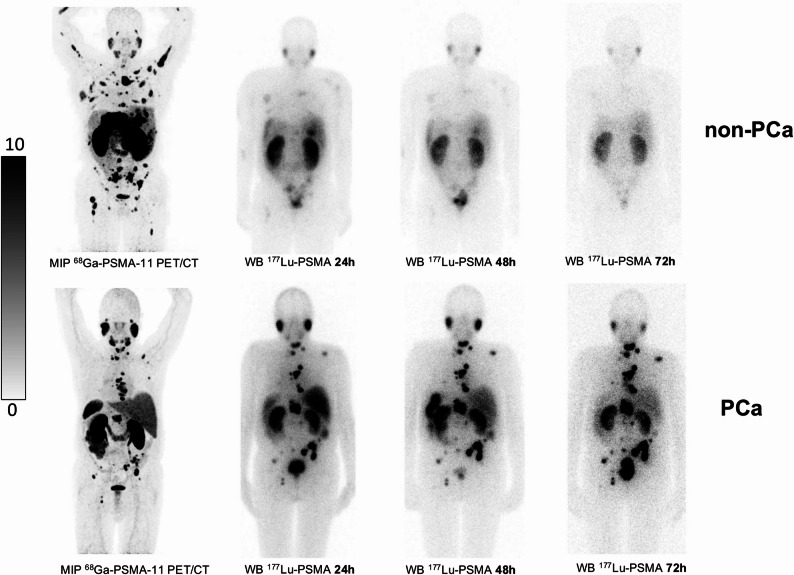
Maximum-intensity projections (MIP) of [^68^Ga]Ga-PSMA-11 PET/CT and posterior [^177^Lu]Lu-PSMA-617 whole-body scintigrams at 24, 48 and 72 h after administration. The top row are images from a patient with HCC (non-PCa, patient No. 3 in Tables 1, 2 and 3) showing rapid tracer washout from tumor lesions, the bottom row are images from a patient with prostate cancer (mCRPC, patient No 7 in Tables 1, 2 and 3) showing the typical retention of tracer over the time

Post-therapeutic serial imaging with [^177^Lu]Lu-PSMA-617 showed similar physiological uptake in both groups (non-PCa and PCa) for salivary and lacrimal glands, kidneys, liver, spleen, intestine and bladder. Representative examples of post-therapeutic [^177^Lu]Lu-PSMA-617 imaging are presented in Fig. 2. Tumor lesions from non-PCa patients showed only low uptake of the radiolabeled PSMA ligand at 24 h p.i., which further decreased with time. Thus, radiotracer uptake could no longer be detected in these tumor lesions at later time points (72–120 h p.i.). In contrast, tumor lesions from PCa patients typically showed intense radiotracer uptake at all time points of post-therapeutic imaging.

### Normal organ dosimetry

For all patients of both groups absorbed dose estimates were calculated considering the following source organs: the liver, the kidneys, the parotid and submandibular glands. The mean source organ absorbed dose per injected activity and the mean half-life of [^177^Lu]Lu-PSMA-617 uptake in respective normal organs for both patient groups are summarized in Table 2 as well as the effective dose in Sv/GBq.

**Table 2:** Absorbed dose per injected activity (Gy/GBq) in normal tissue for all patients. *The effective dose is given in Sv/GBq. ^**†**^Absorbed dose for paired organs is presented as mean between right and left side

Patient	Entity	Effective dose* Sv/GBq	Kidney^†^		Liver		Parotid gland^†^		Submandibular gland^†^	
			eff. T_1/2_ h	D/A_0_ Gy/GBq	eff. T_1/2_ h	D/A_0_ Gy/GBq	eff. T_1/2_ h	D/A_0_ Gy/GBq	eff. T_1/2_ h	D/A_0_ Gy/GBq
1	BC	0.05	23.6	0.65	29.6	0.10	29.0	0.59	29.7	1.03
2	BC	0.04	34.3	0.41	27.2	0.09	31.7	0.42	21.0	0.77
3	HCC	0.02	36.4	0.40	20.1	0.06	26.6	0.40	22.4	0.24
4	RCC	0.08	39.9	0.59	54.8	0.38	61.4	0.81	37.1	1.17
5	AA	0.03	38.3	0.74	19.5	0.08	23.2	0.43	17.2	0.32
6	PCa	0.03	43.5	0.40	20.9	0.06	24.8	0.32	27.1	0.50
7	PCa	0.05	41.7	0.23	21.2	0.06	24.9	0.51	21.4	0.35
8	PCa	0.04	51.5	0.26	45.2	0.11	50.9	0.49	57.9	0.35
9	PCa	0.04	41.9	0.51	34.2	0.04	49.2	1.07	37.1	1.00
10	PCa	0.04	31.4	0.59	19.6	0.05	23.7	0.55	35.2	1.25

The mean effective dose for the non-PCa patients was 0.04 ± 0.02 Sv/GBq and for the PCa patients 0.05 ± 0.03 Sv/GBq, respectively. Comparing non-PCa vs. PCa patients, the following mean absorbed organ dose was observed: 0.56 ± 0.15 Gy/GBq vs. 0.56 ± 0.14 Gy/GBq for the kidneys (*p* = 0.222), 0.14 ± 0.13 Gy/GBq vs. 0.06 ± 0.02 Gy/GBq for the liver (*p* = 0.151), 0.53 ± 0.17 Gy/GBq vs. 0.59 ± 0.28 Gy/GBq for the parotid glands (*p* = 0.999), 0.71 ± 0.42 Gy/GBq vs. 0.69 ± 0.41 Gy/GBq for the submandibular glands (*p* = 0.841). No significant differences for the source organ absorbed dose was observed between both groups. In non-PCa vs. PCa patients, the following mean effective half-life was observed: 34.5 ± 6.5 h vs. 42.0 ± 7.2 h for the kidneys (*p* = 0.095), 30.2 ± 14.4 h vs. 28.2 ± 11.2 h for the liver (*p* = 0.999), 34.4 ± 15.6 h vs. 35.9 ± 13.2 for the parotid glands (*p* = 0.841) and 25.5 ± 7.9 h vs. 35.7 ± 13.9 h for the submandibular glands (*p* = 0.840). With regard to the mean half-life of [^177^Lu]Lu-PSMA-617 uptake in the selected normal organs, no significant differences were observed between the groups of non-PCa and PCa patients (all *p* > 0.840).

### Tumor lesion dosimetry

Post-therapeutic scintigraphy imaging was analyzed for *n* = 18 tumor lesions from the non-PCa patients (range 1–5 lesions per patient) and *n* = 16 tumor lesions from the PCa patients (range: 3–4 lesions per patient). The mean absorbed tumor dose and the respective half-life are presented in Table 3. Significant differences were observed comparing the mean absorbed dose per injected activity of the tumor lesions for the non-PCa and the PCa patients revealing significantly lower values for the non-PCa group (0.49 ± 0.40 Gy/GBq vs. 3.51 ± 2.20 Gy/GBq, respectively, *p* = < 0.001) (Table 3). In addition, the mean effective half-life observed for the tumor lesions was significantly shorter in non-PCa patients compared to the PCa patients (27.1 ± 13.1 h vs. 74.9 ± 23.1 h, respectively, *p* = < 0.001).

**Table 3:** Effective half-life (h) and absorbed dose per injected activity (Gy/GBq) of the target tumor lesions for all non-PCa and PCa patients

Patient	Entity					Tumor lesions		
		Nr.	Localization		SUVmax		eff. T_1/2_ h	Gy/GBq
1	BC	1. 2. 3. 4. 5.	right shoulder left shoulder right hip left hip pelvis (os ilium)		12.4 11.7 17.8 12.6 12.5		29.9 30.6 32.7 33.6 50.0	0.34 0.33 0.74 0.44 0.97
2	BC	1. 2. 3. 4. 5.	right shoulder left humerus right hip left hip left femur		12.5 15.8 9.8 10.2 4.4		21.9 19.5 25.2 17.4 18.8	0.47 0.37 0.18 0.12 0.81
3	HCC	1. 2. 3. 4. 5.	liver left lobe liver right lobe right humerus left femur sternum		31.2 29.8 14.4 17.3 26.9		17.8 10.5 15.9 16.8 18.6	0.32 0.16 0.14 0.13 0.34
4	RCC	1. 2.	Th8 liver left lobe		14.10 11.8		24.9 43.1	1.56 1.18
5	AA	1.	brain		16.8		60.6	0.32
6	PCa	1. 2. 3.	LN supraclavicular left os ilium left femur		25.8 38.8 15.7		72.7 69.5 88.3	8.59 4.91 2.86
7	PCa	1. 2. 3. 4.	sternum right shoulder right os ilium L3		20.3 10.6 10.7 33.4		64.4 63.2 63.5 149.0	2.69 0.91 2.97 2.26
8	PCa	1. 2. 3.	L4 Right os sacrum Right shoulder		73.2 33.4 88.6		81.8 82.3 81.4	8.52 4.58 2.91
9	PCa	1. 2. 3.	Th7 right rip skull base		45.8 38.1 55.3		43.7 73.3 52.9	2.49 1.99 1.43
10	PCa	1. 2. 3.	Th5 L4 Os sacrum		22.8 24.5 22.5		60.6 69.2 82.2	2.69 2.91 3.41

## Discussion

This is the first study to show radiation dosimetry data for [^177^Lu]Lu-PSMA-617 in non-prostate cancer population including hepatocellular, renal cell, and breast cancers as well as anaplastic astrocytoma. Although tracer uptake of non-PCa tumor lesions was intense on [^68^Ga]Ga-PSMA-11 PET/CT, we found a significantly shorter effective half-life for [^177^Lu]Lu-PSMA-617 and consequently a significantly lower absorbed dose per injected activity in tumor lesions of non-prostate cancer compared with prostate cancer.

The role of PSMA as an enzyme is still unclear, even in prostate cancer. PSMA on the prostate cancer cell enables an internalization process that allows endocytosis of bound proteins on the cell surface into an endosomal compartment and thus enables radiolabeled PSMA ligands to accumulate in the cell and deliver their toxic radiation dose in terms of their half-life. Recently published dosimetry data of [^177^Lu]Lu-PSMA-617 and [^177^Lu]Lu-PSMA I&T in prostate cancer showed prolonged retention of ^177^Lu in the tumor lesions resulting in high absorbed doses [20, 28]. In the present study on non-PCa, we observed both, significantly shorter effective half-life (~ 2.5-fold shorter, 27 h in non-PCa vs. 74 h in PCa) and significantly lower absorbed dose per injected activity (~ 7-fold lower, 0.49 Gy/GBq in non-PCa vs. 3.51 Gy/GBq in PCa) in tumor lesions of non-prostate cancer compared with those of PCa. Thus, our results indicate differences in the PSMA biokinetic between non-PCa and PCa tumor cells, reflected by the short retention time and faster clearance of [^177^Lu]Lu-PSMA-617 from tumor lesions of non-PCa. One explanation may be that [^177^Lu]Lu-PSMA-617 is not internalized into the tumor cells of non-PCa. Furthermore, the majority of PSMA expression observed on [^68^Ga]Ga-PSMA-11 PET/CT in non-PCa might be not directly related to the tumor cells themselves, but rather to the tumor-associated neovasculature where internalization may also not occur. Preclinical and translational studies demonstrated functional binding of PSMA-targeted ligands to tumor neovasculature, including in renal cell carcinoma and glioblastoma models [29, 30]. Consistent with this biology, multiple clinical PSMA PET studies have demonstrated tracer uptake in non-prostate malignancies such as renal cell carcinoma, hepatocellular carcinoma, thyroid cancer, salivary gland tumors, and glioblastoma [31–33].

Reports on [^177^Lu]Lu-PSMA-617 in different solid tumors other than prostate cancer mainly consisted of case reports, mostly not presenting dosimetry data. In most cases the clinical outcome was not encouraging. Tolkach et al. reported about a woman with advanced triple-negative breast cancer who was treated with two cycles of [^177^Lu]Lu-PSMA-617 RLT after failure of a variety of systemic therapies [34]. The authors described accumulation of [^177^Lu]Lu-PSMA-617 in tumor lesions on post-therapeutic scintigraphy, but no dosimetry data were provided. This patient showed severe progression four weeks after the second cycle and therefore [^177^Lu]Lu-PSMA-617 RLT was discontinued. Kumar et al. reported on a patient with recurrent glioblastoma multiforme who received 3 cycles of 3.7 GBq of [^177^Lu]Lu-PSMA-617 after failure of resection, radiation and temozolomide treatment. However, again, no dosimetry data have been reported. In the study of Kumar et al., the RLT resulted in significant tumor shrinkage on MRT and improvement of quality of life [35]. Only Hirmas et al. provided dosimetry data for two patients with HCC that received [^177^Lu]Lu-PSMA-617 RLT. Using SPECT/CT dosimetry tumor radiation doses of 0.11 Gy/GBq and 0.02 Gy/GBq were determined, which are at least 10 times lower than those typically achieved by 1 cycle of external-beam radiation therapy of HCC. For this reason, RLT was discontinued after one cycle for both patients [36]. In line with these findings, other case reports on [^177^Lu]Lu-PSMA-617 in metastatic radioiodine-negative thyroid cancer and salivary gland cancer were showing very limited clinical benefit Other case reports on [^177^Lu]Lu-PSMA-617 in metastatic radioiodine-negative thyroid cancer and salivary gland cancer were also showed of very limited clinical benefit [37, 38]. In a prospective, single-center, single-arm study, a different ligand (PSMA-I&T) was used instead of PSMA-617 with [^177^Lu]Lu, and similar dosimetric results were observed in patients with recurrent or metastatic salivary gland cancer (10 patients with adenoid cystic carcinoma and 2 patients with salivary duct carcinoma). J. van Ruitenbeek et al. demonstrated in this study that the absorbed doses to tumor lesions were also low (median 0.07 Gy/GBq, range 0.001–0.63 Gy/GBq) [39]. Although [^177^Lu]Lu -PSMA-I&T was safe and generally well tolerated in salivary gland cancer patients, its efficacy was limited. No objective responses were documented and most patients did not complete the planned treatment cycles due to disease progression or deterioration in general condition, particularly in patients with salivary duct carcinoma.

Although the absorbed doses of the selected normal organs in both groups are in agreement with published data [20, 23, 28, 40, 41], slightly higher doses at least for the kidneys and the liver were observed for non-PCa compared to PCa patients in our study. Even though statistically non-significant, those differences could probably arise from the so-called tumor sink effect known in PCa patients [42]. In PCa tumor lesions compared to normal tissue, much higher PSMA expression and uptake of radiolabeled PSMA ligands is often observed. For this reason, high tumor burden may lead to high radiotracer uptake in tumor tissue and consequently to lower radiotracer uptake in normal organs resulting in reduced radiation exposure of non-tumor tissue (normal organs). In our study, only the first cycle RLT in PCa patients was included. For these patients, who often have high tumor volume, the tumor sink effect is more likely to be observed than in patients who already received several RLT cycles or have low tumor volume.

The higher absorbed dose observed for the liver in non-PCa compared to PCa patients in our study (0.14 ± 0.13 Gy/GBq vs. 0.06 ± 0.02 Gy/GBq) could be explained by the high individual absorbed dose in one patient (RCC) of 0.38 Gy/GBq. Although liver function was normal in this patient, she had moderate renal insufficiency (estimated GFR 19 ml/min) after tumor nephrectomy, which may result in slower clearance of circulating [^177^Lu]Lu-PSMA-617 and therefore may cause prolonged circulation and, thus, a higher absorbed dose in the liver.

Considering the heterogeneity of PSMA expression in different non-PCa entities, it would be an interesting approach to further evaluate the potential of [^177^Lu]Lu-PSMA-617 RLT by a pre-therapeutic PET-based dosimetry estimation using PSMA-specific radiotracers in combination with long-lived radionuclides such as ^89^Zr (half-life 3.27 d) [43]. This may allow an improved selection of patients who could benefit from [^177^Lu]Lu-PSMA-617 RLT. In addition, due to the rapid washout of the tracer from the tumor environment of non-PCa, therapeutic radiopharmaceuticals with shorter half-lives and higher energies (e.g., ^90^Y with a half-life of 2.7 d or ^67^Cu with a half-life of 2.6 d) may be more appropriate and may have higher efficacy. These issues should be evaluated in further studies.

### Limitation

There are important limitations to this study. First, this is a retrospective analysis comprising only a small and heterogeneous patient population. Second, for dosimetry we used our standard protocol starting with the first imaging at 24 h p.i, which might be not appropriate in non-PCa due to the rapid washout from tumor lesions. Another limitation of this study is the dosimetry protocol, which uses 2D dosimetry (whole-body scan) instead of the most advanced 3D dosimetry (SPECT or SPECT/CT). However, the use of 2D dosimetry with planar images alone tendes to overestimate the absorbed dose compared to the more accurate 3D dosimetry (SPECT) [44]. Taking this into account, the absorbed dose calculated with 3D dosimetry using SPECT (or SPECT/CT) would be even lower in the non-PCa group, confirming our finding that [^177^Lu]Lu-PSMA-617 RLT does not appear to be a suitable treatment option for non-PCa. The non-standardized administered activities present another limitation. In fact, low activity of [^177^Lu]Lu (e.g., 400 MBq) would be appropriate for dosimetric purposes. However, the intention of this study was not only to collect dosimetric data on [^177^Lu]Lu-PSMA-RLT in non-PCa patients, but also to achieve an antitumor effect in these non-PCa patients who had exhausted all other treatment options. The [^177^Lu]Lu activities used were based on individual characteristics such as the patient’s condition, tumor burden, tumor dynamics, and safety aspects such as pre-existing hematotoxicity. Based on these disappointing results in non-PCa patients (rapid clearance of [^177^Lu]Lu-PSMA-617 from tumor lesions and low absorbed doses in tumor lesions in non-PCa), no additional cycles were administered. PCa patients, however, received their planned 4–6 cycles of [^177^Lu]Lu-PSMA-617.

## Conclusion

Radioligand therapy with [^177^Lu]Lu-PSMA-617 does not appear to be a suitable treatment option in patients with non-prostate cancer. Despite the high tumor uptake in [^68^Ga]Ga-PSMA-11 PET/CT screening, the effective half-life of [^177^Lu]Lu-PSMA-617 in tumor lesions of non-PCa patients was significantly shorter than of PCa patients resulting in lower absorbed doses per injected activity.

## Data Availability

The datasets generated during and analyzed during the current study are available from the corresponding author on reasonable request.
